# Decellularization Protocols for Esophagus Bioengineering: A Systematic Review

**DOI:** 10.3390/bioengineering12121292

**Published:** 2025-11-24

**Authors:** Alexandre Goussens, Patricia Renard, Alexandra Dili, Louis Maistriaux, Julia Vettese, Marie Longton, Benoit Lengelé

**Affiliations:** 1 Morphology Lab (MORF), UCLouvain—IREC (Institut de Recherche Expérimentale et Clinique), Avenue Emmanuel Mounier 52-B1.52.04, 1200 Brussels, Belgium; alexandre.goussens@uclouvain.be (A.G.);; 2 Department of Gastrointestinal Surgery, CHU UCL Namur Site Godinne, 5530 Yvoir, Belgium; 3 Unité de Recherche en Biologie Cellulaire (URBC), Namur Research Institute for Life Sciences (NARILIS), University of Namur (UNamur), 5000 Namur, Belgium; 4 Laboratory of Hepato-Gastroenterology, Institut de Recherche Expérimentale et Clinique, Université Catholique de Louvain, 1200 Brussels, Belgium; 5 Bibliothèque des Sciences de la Santé (BSS), Université Catholique de Louvain, 1200 Brussels, Belgium; 6 Department of Plastic and Reconstructive Surgery, Cliniques Universitaires Saint-Luc, 1200 Brussels, Belgium

**Keywords:** decellularization, esophagus, esophagectomy, bioengineering, systematic review, Sodium Dodecyl Sulfate, Sodium DeoxyCholate

## Abstract

Background: Numerous protocols exist concerning the decellularization of the esophagus, a potential alternative to the classical surgical approach for the reconstruction of the digestive tract after esophagectomy. This systematic literature review (SLR) aimed to provide an overview of the effectiveness of the current protocols. Methods: This SLR was conducted in PubMed, EMBASE, and Scopus until September 2025. Study selection, data extraction, and quality assessment were performed by two independent reviewers according to the inclusion/exclusion criteria. Results: A total of 2494 references were screened after removing duplicates. Among these references, 26 articles were included. The large majority of studies (24/26) used Sodium Dodecyl Sulfate (SDS) or Sodium DeoxyCholate (SDC), and the most common physical method was the cannulation of the esophagus (17/26). The animal model was very heterogenous. All protocols except one showed no residual cell nuclei, with only 5/19 papers confirming a satisfactory residual amount of DNA. The assessment of the extracellular matrix (ECM)—mostly qualitative—revealed global preservation but with a systematic loss of glycosaminoglycans (GAGs). Conclusions: The decellularization of the esophagus is feasible, but the definition of the optimal protocol to achieve this goal remains difficult because of the important heterogeneity among the different studies.

## 1. Introduction

The incidence of esophageal neoplasia is currently increasing worldwide and more patients will need esophageal excision and reconstruction [1]. There is a clinical need to improve post-operative outcomes in esophageal surgery, with very broad indications. Usually, the reconstruction of the digestive continuity is achieved by transposition of an autologous segment of healthy stomach, colon, or small intestine. These current reconstructive solutions, however, are not optimal due to poor functional results, even in the long term, resulting in a significant impairment of quality of life [2]. This complex surgery presents high morbidity and mortality rates, even in expert centers [3]. The increase in the number of bariatric operations around the world, altering the stomach, which is the best choice for reconstruction after esophagectomy, requires even more complex reconstructions [4,5].

The creation of an esophageal graft by tissue engineering (TE) could therefore offer an alternative option to the usual methods for challenging cases in which the gastro-intestinal tract has been resected. Thereafter, if demonstrated to be reliable, it could become a standard of care, improving post-resection outcomes and decreasing complications. It could also avoid the sacrifice of healthy abdominal organs for the reconstruction and preserve the normal anatomy. The ideal substitute should be sterile, biocompatible, noncytotoxic, and keep biomechanical properties similar to the native tissue. Moreover, the esophagus has some particularities: the presence of saliva, microbiota, food, and gastric acid in the lumen, as well as a deformation of its caliber after deglutition.

Many devices have been tested such as synthetic, naturally derived, or decellularized scaffolds. Synthetic scaffolds have the advantage of being reproducible, low-cost, sterile, and easily available. In contrast, they are poorly biocompatible due to a hydrophobic surface preventing cell attachment. They may release some toxic products by degradation, and they induce foreign body reactions. After some attempts, they are not used alone anymore because of a high rate of anastomotic leakage and stricture [6,7,8]. The application of natural proteins such as collagen enhances the surface cell adhesion but complicates the manufacturing process. When applied alone (with a transient stenting support), naturally derived scaffolds lead to strictures [9,10]. The elaboration of a decellularized scaffold is challenging in terms of reproducibility, absence of immunogenicity, and preservation of the extra cellular matrix (ECM). Several decellularized matrices (DM) are already used for clinical or experimental purposes, such as for the heart [11], coronary artery [12], heart valve [13], skin [14], liver [15], trachea [16] and small intestine [17]. The residual ECM is biodegradable and biocompatible even if xenogeneic [18]. It contains native proteins and growth factors but also the native three-dimensional ultrastructure, which plays a role in biomechanical properties and cell adhesion. DM can also stimulate vascular ingrowth, cellular proliferation, and differentiation, while reducing the inflammatory process of immune reaction [19,20,21,22]. Some studies have shown that site-specific ECM seems to favor tissue-specific cell phenotypes and proliferation [23,24].

Several esophageal decellularized patches have been successfully used in clinical practice for mucosal or small transmural defects [25,26,27,28], but repairing a circumferential full-thickness esophageal segment remains the principal challenge that cannot be overcome yet. To date, only four preclinical trials used a circumferential decellularized esophageal graft in a pig or rabbit model with mitigated results [29,30,31,32]. Optimizing the decellularization protocol is a major part of esophagus bioengineering success. Confirming the growing interest in this field, there is already a clinical case report of the use of a tissue-engineered esophageal graft [33].

Numerous teams used different decellularization protocols for the esophagus, but no review exists to compare their different results. The aim of this systematic review is to provide a descriptive summary of the current results concerning the decellularized esophagus, with a focus on decellularization effectiveness and the ECM preservation. Moreover, this review points out the limitations of existing research and highlights areas for future investigations.

## 2. Materials and Methods

The guiding question for this systematic review was formulated according to the PICO format (Patient/Problem, Intervention, Comparison, Outcome(s)): Which protocol of decellularization (I) of the entire esophagus (P) has the best results in terms of cell removal but also in the preservation of the extracellular matrix (O), compared to native controls (C) [34]?

This systematic review was registered on PROSPERO (CRD42024507630) and was conducted according to the Preferred Reporting Items for Systematic Reviews and Meta-Analyses (PRISMA) guidelines [35].

### 2.1. Eligibility Criteria

This review includes original peer-reviewed studies based on the following inclusion criteria: (A) publication in an English-language journal [36]; (B) a study containing the decellularization protocol of an esophagus with a native control group no matter the species, and (C) the results of the decellularization process according to at least one of Crapo’s criteria [37]. The exclusion criteria included: (A) letters, editorials, review articles, conferences, patents, and meeting abstracts; (B) studies reporting the decellularization of patches or another segment of the esophagus other than a full-thickness circumferential esophagus.

### 2.2. Literature Search Strategy

Literature searches were conducted on 6 February 2024, with a renewal on 8 September 2025 in Embase, PubMed, and Scopus as sources for published literature. The following search concepts were used for the literature search: “esophageal” AND “decellularization”. No publication date restrictions were applied. The full research strategy is shown in Table 1. In addition, the electronic search of the databases was complemented by a manual search in the reference lists of included articles for additional studies not retrieved by our initial search. Experts in the field were contacted for any missing reference.

### 2.3. Study Selection and Data Collection Process

The articles were initially screened and included based on their titles and abstracts following predefined criteria. Then, the full text of each selected article was analyzed separately by two authors to define the relevance of the work. The collaboration platform Rayyan was used [38]. Articles were reviewed, evaluated, and discussed by the authors to resolve discrepancies. A standardized, pre-piloted form was used to extract data from the included studies for assessment of study quality and evidence synthesis. Extracted information included the following: author(s), year of publication, species, age, sex, weight, detailed protocol of decellularization and detergent(s) used (including duration, temperature(s), concentration(s)), sterilization technique, result of the decellularization according to the Crapo’s criteria [37], composition of the ECM, biomechanical test, cytotoxicity and cytocompatibility (or biocompatibility) test, and in vivo transplantation. Outcomes considering a modified decellularized scaffold during the process (e.g., using crosslinking agents) were not taken into account. When missing data were present, we contacted the authors. For not reported quantitative data, we used the “a ruler for windows” software to extract measurements directly from the graphs.

### 2.4. Quality Appraisal

The methodological quality and risk of bias were assessed by the Office of Health Assessment and Translation (OHAT) tool, which seems to be the most appropriate to evaluate studies concerning in vitro experiment of the esophagus [39]. Several biases are evaluated such as selection, confounding, performance, attrition/exclusion, detection, and selective reporting bias. Two reviewers independently rated each domain as presenting a certainly low, probably low, certainly high, or probably high risk of bias, as indicated in the OHAT guidelines. Any discrepancies were resolved through discussion.

### 2.5. Data Analysis

Because the scope of this review was large in terms of outcome measures, a systematic review was performed instead of a meta-analysis.

## 3. Results

### 3.1. Study Selection

**Figure 1:**
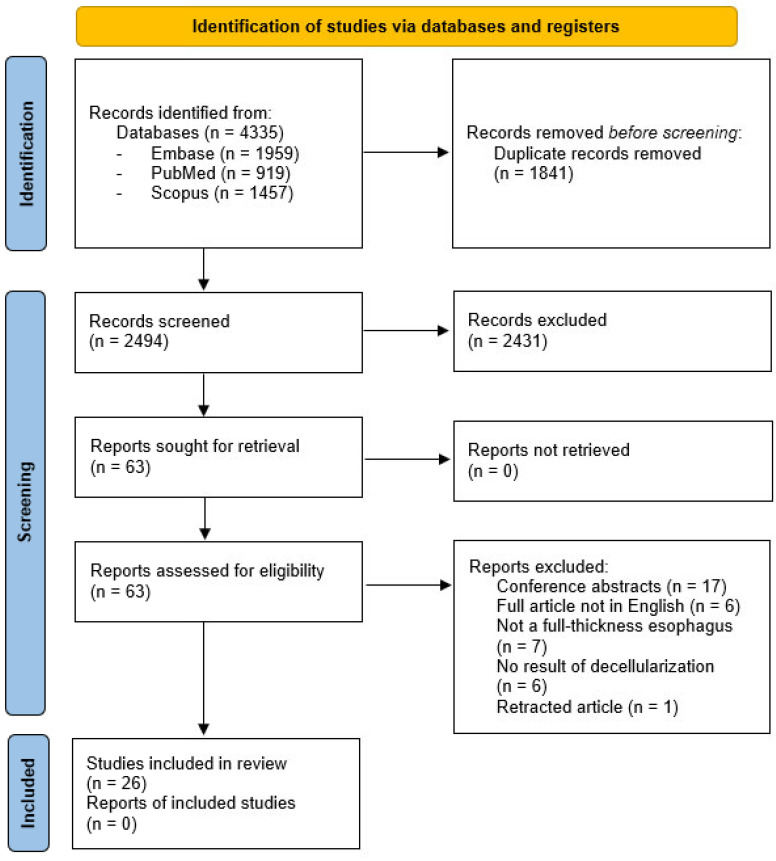
PRISMA flow diagram [35].

A total of 4335 articles were initially retrieved from electronic search: 1959 papers from Embase, 919 papers from PubMed, and 1457 from Scopus. After duplicate removal, 2494 publications remained and were screened with their title and abstract. Following the exclusion of 2431 articles, 63 full-text articles were assessed for eligibility. Finally, 26 studies were included in this systematic review (Figure 1).

### 3.2. Study Characteristics

Detailed data from the 26 included publications are presented in Table 2 and Table 3. In the 26 articles, 2 used human esophagi [40,41] and 24 used animal esophagi: big mammals (12 pigs [21,29,30,31,32,42,43,44,45,46,47,48], one goat [49] and one macaque [50]) and small ones (eight rats [51,52,53,54,55,56,57,58] and two rabbits [59,60]). There is no information on the age of the human cadaveric donors, but it ranges from one day to six months for the animal models (immature and mature models).

The large majority (24 of the 26 studies) used Sodium Dodecyl Sulfate (SDS) or Sodium DeoxyCholate (SDC) as detergents to perform the decellularization. Only two authors used other decellularization solutions: Soapnut Pericarp Extract (SPE) [49] or a comparison of two protocols, deoxycholic acid (DEOX) versus Triton X-100 [51]. A concentration of 4% was used for all studies using SDC, while the concentration ranged from 0.002 to 5% for studies using SDS.

**Figure 2:**
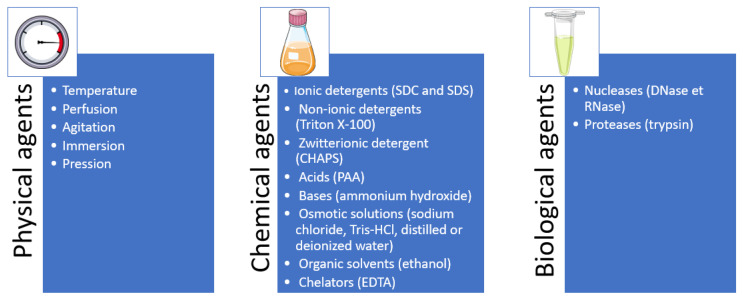
Summary of the decellularization methods for the esophagus.

The most common physical method was the intraluminal cannulation of the esophagus (17 protocols, 65%) but bathing (12%) [45,47,57], immersion with agitation (19%), [40,49,51,52,53] and perfusion (4%) [60] were also used. The median duration of the decellularization protocols was 97.5 h (8.5–395 h), with 10 protocols lasting 72 h or less. Four protocols were completed at room temperature (RT) [29,30,49,56], but eleven required two different temperatures (4 °C, RT or 37 °C), four required three different temperatures (4 °C, RT, 30 °C, or 37 °C), and seven were not informed. Furthermore, five protocols used the freeze/thaw process as a physical agent of decellularization but without nitrogen (snap freezing) and from temperatures of −30 °C to −80 °C [31,40,43,46,50]. In only five studies, the esophagi benefited from a strict sterilization process with gamma irradiation (1.8 to 25 kGy) or Ethylene Oxide [29,30,46,47,54], while the others were treated with antibiotics, antimycotics, peracetic acid, ethanol, sodium azide, or nothing. Figure 2 summarizes all decellularization methods used for the esophagus.

All the protocols of decellularization succeeded to eliminate the cell nuclei according to histological assessment (DAPI and/or H&E), except one where no histological results were provided [56]. Only 19 authors measured the residual dsDNA amount from which 5 confirmed an amount inferior to 50 ng per mg of dry tissue [30,31,41,44,60]. The exploration of the different ECM components preservation—as the collagen, laminin, elastin, fibronectin, or glycosaminoglycans (GAGs)—was often qualitative with a global retention. On the protocols exploring these components, 6/17 were quantifying for the collagen (35%), 1/10 for the laminin (10%), 3/11 for the elastin (27%), 1/10 for the fibronectin (10%), and 10/13 for the GAGs (77%). The GAGs and the fibronectin were the only ones to show a systematic decrease, while the others revealed heterogeneous results.

Fourteen authors explored the biomechanical characteristics of the DM, with only four using a tubular scaffold. They noted a stiffer matrix after decellularization but more circumferentially deformable.

On all of the 26 esophageal decellularization protocols, only 16 benefited from a cytotoxicity test (62%) and 12 from a biocompatibility test (46%). Six studies tested the graft of their decellularized esophagus.

### 3.3. Risk of Bias Assessment

**Figure 3:**
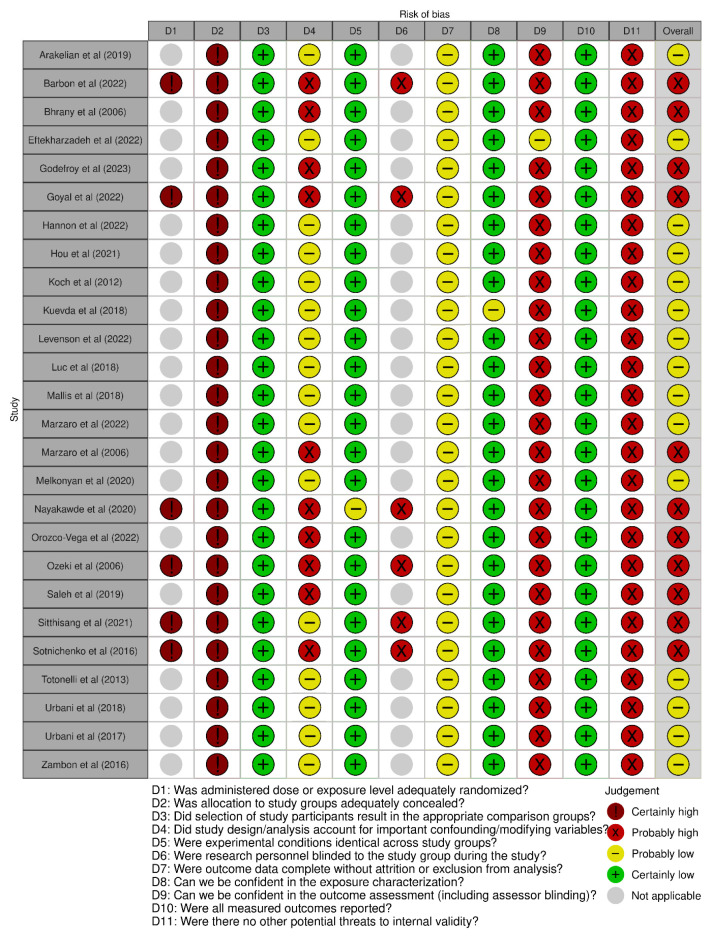
Risk of bias [29,30,31,32,40,41,42,43,44,45,46,47,48,49,50,51,52,53,54,55,56,57,58,59,60,63].

**Figure 4:**
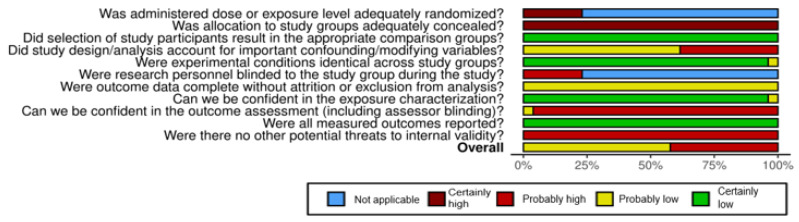
Risk of bias summary.

Figure 3 and Figure 4 summarize the risk of bias in the included studies [62]. Regarding selection biases, there was no randomization between the control group and the experimental one or between the different experimental groups when it was applicable. On the other hand, each protocol included in this review was compared to an appropriate native esophagus. Some confounding biases exist in 10/26 studies, since some important data were missing such as the age, weight, or time since the death of the animal model. Almost every study showed identical experimental conditions except one [43]. There seemed to be no attrition or exclusion biases, but for the detection biases, only one article described blinding for two pathologists in the classical histological analysis (not specified for the DNA quantification) [58]. The characterization of the protocols was well described except for one (no timing nor dosage for some steps) [57]. All of the announced outcomes were reported (no selective reporting biases). Other potential biases could be present: sample selection bias, bias related to the reproducibility of results (linked to a small number of samples), or to the insufficient standardization of the decellularization protocol (technical variability in a different step of the protocol). Moreover, the absence of independent replication of these protocols, which is common in this emerging field, may also limit the overall reproducibility and increase the risk of bias.

Overall, we considered 15 of the 26 studies with a probably low risk of bias and the others with a probably high risk of bias according to the OHAT tool. Attention was paid to the presence of confounding variables and attrition/exclusion biases to settle them. These results may be explained by the exploratory and experimental nature of the field. However, excluding such studies could limit access to the relevant data and methods. This is why their potential biases were transparently acknowledged and taken into account, permitting their retention to ensure the comprehensive analysis of the subject.

## 4. Discussion

The aim of this review was to summarize the effectiveness of all the protocols of decellularization of the esophagus with a focus on the criteria of Crapo et al. (2011) [37]. The attention was focused on pertinent data providing evidence of more efficient outcomes for TE purposes.

### 4.1. Decellularization Effectiveness

The decellularization of tissues and organs to produce ECM scaffolds requires a balance between maintaining the native ECM structure and the removal of the immunogenic materials such as DNA, mitochondria, membrane lipids, and cytosolic proteins. These remnant cellular components can generate an adverse inflammatory response conducive to poor constructive remodeling [37,64]. Crapo et al. (2011) [37] established three criteria—which are commonly used—with the purpose of defining quantitative metrics on a decellularization standard: <50 ng dsDNA per mg ECM dry weight; <200 bp DNA fragment length; and lack of visible nuclear material in tissue sections stained with 4′,6-diamidino-2phenylindole (DAPI) or H&E (Hematoxylin and Eosin). Nevertheless, these thresholds are quite arbitrary and not standardized, especially when considering the mechanisms of DNA recognition by host immune cells are still incompletely understood. They also could not be appropriated to all species or organs [61,64,65,66].

However, all authors do not use these criteria, and simpler proof of decellularization can be seen as the absence of positive staining for intact nuclei at a histological level or as a significant reduction in the dsDNA amount compared to native samples. In this review, all protocols achieved the absence of observable cell nuclei at the histology except Melkonyan et al. [56]. Only five protocols reached an amount of dsDNA lower than 50 ng dsDNA/mg of ECM dry weight, using SDS [41,44,60] or SDC [30,31]. This mainly concerns big mammals (three pigs and one human) but also a small one (rabbit). Even if the threshold was not reached, 14 other protocols obtained good results, with a significant decrease in the amount of dsDNA [29,40,42,43,46,47,49,51,53,54,55,56,59,63]. It is noteworthy that five of these protocols reached an amount of dsDNA lower than 50 ng but on wet tissue, which therefore suggests a higher rate on dry tissue [40,47,49,55,63].

Three authors explored the immunogenic potential of their ECM by searching the MHC (Major Histocompatibility Complex) class I and/or II and confirmed its absence [40,45,60]. If the search for the Human Leukocyte Antigen (HLA) class I (equivalent to the MHC class I)—a ubiquitous cell surface protein—is always performed in case of clinical allotransplantation in humans, then this is not the case in TE. Therefore, major interest in adding the quantification of this item to define an effective decellularization has been raised recently in the field of human TE methods [67].

### 4.2. Esophageal Origin

ECM constituents are highly conserved across species, but there are major differences when decellularizing organs from small or large animal models. First, the quantity of detergent, its concentration, and the duration of the process are higher in large animal models. Big mammals are more expensive but have the advantage of being anatomically closer to humans. In terms of size, anatomy, histology (e.g., more smooth muscle in the lower esophagus) and biomechanical properties, the porcine esophagus is quite similar to a human’s [68,69]. On the contrary, the esophagus of the rodents is composed exclusively of skeletal striated muscle. On the other hand, smaller animals allow for a reduction in costs and a larger number of samples/replicates. Other important factors have to be considered such as the breed and age of the animal model or the segment of the harvested esophagus (cervical or distal). The age can influence cell binding sites and phenotypes, the ECM degradation rate, or the biomechanical properties [66,68]. Tottey et al. demonstrated differences with age in pig small intestinal submucosa (SIS) ECM, with a slower degradation rate, a higher elastic modulus, and less GAGs in the older pigs [70]. The choice of age model must then be adapted to the aim of the research. The rodents are classically mature for the size of the animal model. On the other hand, only 3 of the 12 porcine models were mature because of the difficulty in handling adult pigs weighing more than 50 kg, while the esophageal size is amply sufficient [31,44,46]. Two authors already explored the human esophagus, considering the possible future clinical implications.

### 4.3. Decellularization Methods

**Figure 5:**
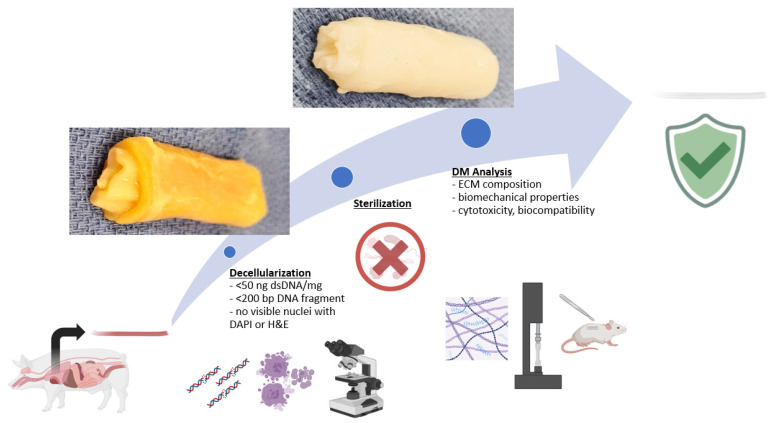
Summary of the creation of a decellularized matrix (using Biorender).

The optimal decellularization protocol must be tailored to the specific biochemical and physical characteristics of the native tissue: the cellularity, density, lipid content, and thickness. This process usually involves a combination of physical, chemical, and enzymatic agents to remove all of the cells while preserving the ECM. Indeed, every strategy of decellularization includes an alteration of the ultrastructure and the composition of the ECM, while it is expected to be minimal. It consists of a minimum of three major steps: decontamination, application of detergent(s), and rinsing and removal of the residual detergent and cells/ECM debris. If the DM will be used for clinical purposes, it also must be sterilized (Figure 5).

The esophagus is a thick tube composed of four concentric layers: mucosa, submucosa, muscularis externa, and adventitia. The mucosa consists of a stratified squamous epithelium serving as a barrier against the luminal content (microorganisms, food, saliva, gastric acid, and air). The submucosa is composed of a loose connective tissue with blood vessels, nerves, and small glands for the lubrification of the tube, enhancing food transit. The muscularis externa is formed by two layers of muscle—an inner circular and an outer longitudinal—containing a mix of striated and smooth muscle cells. This muscle coating allows coordinated contractions known as peristalsis. The adventitia is a classically loose connective tissue attached to neighboring tissues.

The ideal esophageal substitute should have biocompatibility, internal resistance to the content of the lumen, and adequate mechanical characteristics to allow food to pass through. Moreover, the DM should have properties allowing for future recellularization, including cell adhesion, infiltration, and proliferation, and avoid inflammation or infection.

#### 4.3.1. Physical Agents

**Temperature**—Freeze/thaw cycles can lead to cell disruption and lysis by the formation of intracellular ice crystals. However, this cannot remove the subsequent residues and thus typically needs chemical and/or biological agents afterward. Burk et al. demonstrated more effective decellularization when using freeze/thaw cycles with detergents than detergents alone in an equine large tendon model [71]. It is particularly useful in thick tissues as a first decellularization step. In this review, five studies used a freeze/thaw process involving big mammals (three pigs, one macaque, and one human), because this process is expected to reduce the quantity and the time exposed to detergents [31,40,43,46,50].

**Perfusion**—The perfusion of detergents through the intrinsic vascular system is an effective route to the whole organ/tissue due to the natural proximity of the cells to the vascular network for nutrients and oxygen. Direct vascular access can allow for a lower detergent concentration and exposure time, therefore limiting extra damage to the ECM [72,73]. However, such a major vascular pedicle does not exist in the esophagus. Indeed, the esophagus only benefits from tiny vascular branches that cannot be practically employed. Despite this limitation, Hou et al. achieved the only perfusion protocol for the decellularization of the cervical esophagus in a rabbit model [60]. They used the common carotid artery, even if this vascular access is not selective, and decellularized the contents of the neck (cervical esophagus, pharynx, larynx, trachea, and thyroid). Then they isolated the decellularized esophagi among the other organs. Their protocol was effective with a short duration (65 h) and a low dsDNA residue (41 ± 7 ng/mg). The efficiency of the perfusion protocol is determined by the perfusion route and mode (arterial versus vein; antegrade versus retrograde) as well as the perfusion pressure, temperature, and flow rate. There is also an important impact of the types and concentrations of perfusate and the dimension of the organ/tissue [74]. Up to now, despite the fact that only perfusion decellularization protocols lead to the complete cell and allogenic clearance of organs, unfortunately, no model has been described as overcoming the segmental blood supply of the esophagus, especially in large animals close to humans.

**Immersion and agitation**—This is a simple and reproducible way to decellularize the thin or small tissues/organs by submerging the sample in the decellularization solution with a constant mechanical agitation. The main obstacle to this technique is the important thickness and density of the organ/tissue limiting the diffusion of detergent, requiring longer exposure time, and then provoking more damage on the ECM [64,74]. Nevertheless, this is an effective alternative when there is no similar vascular access as for the esophagus. Seven authors used this process for decellularization, of which two used an immersion process without agitation [45,47]. In more than half of the cases, the total protocol duration exceeded three days, with the longest protocol of the review exceeding two weeks (395 h) [45]. Immersion and agitation protocols were essentially the first protocols performed in esophagus decellularization with the four oldest papers using it [45,47,51,52].

**Pression**—One way to improve the effectiveness of decellularization is to create a pressure gradient across the tissue. This can drive the decellularization agents through the tissue and remove cell residues of the ECM [64,74]. This is particularly suitable for the esophagus—a hollow organ—that can be cannulated. For this reason, it is not surprising that 18 out of the 26 reviewed protocols used this method. The big mammals mostly benefited from the cannulation because of a thick and dense esophagus. This was the case for 10 out of 12 pigs’ esophagi, the macaque’s one, and one of the two humans’ esophagi [29,30,31,32,41,42,43,44,46,48,50,63]. Since the goal here is not to mimic the physiologic conditions, an important heterogeneity of pressure can be observed, ranging from 0.1 to 70 mL/minute. In the absence of a selective perfusion model, cannulation seems the optimal physical way to decellularize the esophagus.

#### 4.3.2. Chemical and Biological Agents

As previously said, the choice of decellularization agent must be tailored to the characteristics of the organ/tissue, and there will always be a degree of alteration of the ECM. The main objective is to minimize these alterations while performing an effective decellularization, knowing that the effect of the detergent increases with the exposure time. Chemical treatments disrupt cellular and molecular bonds and remove cellular residues, while biological treatments—typically enzymatic agents—bring high specificity for the withdrawal of cell components and/or undesirable ECM constituents by hydrolyzing specific molecules within cells or cell–matrix adhesions. Usually, biological treatments are employed when chemical ones cannot reach effective decellularization by themselves [37,64,74]. The chemical agents include ionic detergents (SDC and SDS), non-ionic detergents (Triton X-100), zwitterionic detergent (Cholamidopropyldimethyl Ammonio Propane Sulfonate (CHAPS)), acids (peracetic acid (PAA)), bases (ammonium hydroxide), hypertonic solutions (sodium chloride), hypotonic solutions (Tris-HCl, distilled or deionized water), organic solvents (ethanol), and chelators (Ethylenediamine Tetraacetic Acid (EDTA)). For the biological agents, it essentially concerns nucleases and proteases (such as trypsin).

**Osmotic agents**—Hyper- or hypotonic solutions decellularize tissues through osmotic effects, leading to dehydration, lysis, and death of the cells. These solutions can also effectively remove residual chemicals and cellular debris but can lead to the dispersion of antigens in tissues. Classically, they are not sufficient for effective decellularization [74,75]. In this review, all of the authors used at least one osmotic agent in their protocols, except for the one of Saleh et al., where it is not reported [55]. Only four protocols used hypertonic solution alone [31,47,51,53], while twenty-one used a hypotonic solution or a combination of the two [42,45,48,54,59]. Since these solutions are easily washed out of the tissues, the possible cellular toxicity is weak, making them a good choice for rinsing solutions while helping with decellularization.

**Ethylenediamine Tetraacetic Acid**—EDTA is a chelating agent that binds divalent metal cations required for several adhesion molecule activities, causing cell–cell and cell–ECM dissociation. EDTA is also an inhibitor of metalloproteases. Chelating agents alone are insufficient for cell removal, even with the use of physical methods [37,64]. Eleven protocols used EDTA on top of a large variety of detergents [32,40,41,46,49,50,51,52,53,56,63].

**Sodium DeoxyCholate**—SDC, such as other detergents, is a soluble amphiphile molecule that can disrupt hydrophilic and hydrophobic interactions, solubilize cell membranes, and dissociate DNA from proteins, but also disrupt covalent bonds between proteins in the ECM. This results in an elimination of cells and genetic contents but at the cost of the risk of alteration in the decellularized ECM scaffold and reduced mechanical strength [29,49,52]. SDC already showed effectiveness in protocols of decellularization in such organs as the heart [76], trachea and lungs [77], pancreas [78], or diaphragmatic tissue [79]. In this review, 14 authors used the 4% SDC (always at this same concentration) and 2 reached an amount of dsDNA < 50 ng/mg [30,31]. It is interesting to note that every protocol with 4% SDC required an extra step with nucleases—DNase-I for each protocol but also RNase-A for the protocols of Orozco-Vega et al. (2022) and Bhrany et al. (2006) [46,52]. All decellularization processes with SDC also used osmotic solutions. Five protocols were quite similar, because they originated from the same group of P. De Coppi and systematically used rinsing, 4 h of 4% SDC, and 3 h of 2000 KU of DNase at RT with cannulation [30,42,48,54,59]. The only minor differences concerned the rinsing solution (deionized water with or without sodium chloride) and the number of cycles/total duration, because they were performed with different animal models (rat, rabbit, and pig). Other teams used this type of biochemical protocol [29,31,40,43,45,57] or with the addition of EDTA [50,56]. Of the 14 protocols, 10 used only the RT or maximum two different temperatures, simplifying their implementations. Orozco-Vega et al. (2022) employed a complex sequence of 1% Triton X-100, ammonium sulfate, 4% SDC, DNase-I, RNase-A, and EDTA with different durations depending on the age of the pigs [46]. Nayakawde et al. (2020) [43] compared three protocols in a pig model: the 4% SDC + DNase-I; a combination of 6% Tri-n-Butyl phosphate (TnBP, an organic compound that disrupts protein–protein interactions), 6% Triton X-100, and DNase-I; and ultrasonication (physical method) and DNase-I. Only the first protocol with SDC achieved adequate decellularization. In this study, they quantified the SDC residual content in the decellularized matrix after a 12 h exposure and found a negligible quantity of SDC (0.009%), concluding that there is a low risk of accumulation in the tissues [43]. It is noteworthy that, of the 14 protocols using SDC, 10 concerned big mammals (eight pigs, one human, and one macaque). This explains why physical methods were often used such as cannulation (12 protocols) or freeze/thaw cycles (5 protocols). This also contributes to a longer duration of decellularization: 21–62 h in the rat and rabbit versus 24–395 h in big mammals. Globally, 6 of the 14 protocols were considered short, with a duration inferior to 72 h.

**Sodium Dodecyl Sulfate**—SDS is also an ionic detergent like the SDC but more commonly used in the literature. It is widely used in numerous organs or tissues such as the heart [11], cardiac valves [80], the lung [81], the liver [82,83], the kidney [82,84], the skin [85], the bone [86], the fascia lata [87], the ear [88], and the face [89]. As previously described, ionic detergents may have harmful impacts on the ultrastructure of the ECM, and this is particularly true for SDS, which has a strong interaction with ECM proteins. For this reason, short exposure times and low concentrations are preferred. It also needs several rinsing cycles to ensure the correct removal of residual SDS, which may induce cytotoxicity during recellularization. Nevertheless, it is very efficient in nuclear and cell removal [37,64,74,75,90]. In this review, 11 authors used SDS at different concentrations ranging from 0.002% to 5%. Three protocols achieved the threshold of an amount of dsDNA inferior to 50 ng/mg [41,44,60]. Only two teams used SDS alone [44,55], while all of the others combined it with Triton-X100 [47,52,58], nucleases [32,41,47,52,63], CHAPS [53], or ammonium hydroxide [40]. All protocols used osmotic agents except three [41,47,55]. Three protocols were performed by the same team of L. Arakelian et al. and consisted of decontamination, 72 h of 2% SDS, and 3 h of DNase-I by cannulation in the pig or the human esophagus under sterile conditions [32,41,63]. Of the eleven protocols, they all needed, at some point, an adaptation of the temperature, with four protocols even requiring at least three different temperatures [31,32,40,52]. Hammouda et al. showed the impact of temperature on SDS, which precipitates below 15 °C while its solubility increases with the temperature [91]. Increasing the temperature could enhance the solubilization of SDS and facilitate its diffusion in the tissues but also its removal. However, high temperatures should be avoided due to the risk of further protein denaturation [41]. On the contrary, a low temperature could decrease the penetration of the SDS and retain SDS precipitates in the tissues. The total duration of SDS-based decellularization protocols, essentially impacted by the need for important rinsing, ranged from 8.5 to 228 h. There was an important variation both in small mammals (8.5–217.5 h) and in big ones (144–228 h). Only three protocols were considered short—inferior to 72 h—and concerned small mammals (two rats and one rabbit) [53,55,60]. Cebatori et al. demonstrated that the abundant rinsing of decellularized cardiac valves to reach a residual SDS concentration in the washing liquid under 50 mg/L (or 0.005%) was permitted to avoid cytotoxicity in human endothelial cells [92]. Godefroy et al. used an ion-exchange resin or an activated charcoal cartridge to optimize the SDS removal of their esophageal DM. They achieved an excellent result with low residual SDS in the washing solution (0.27 ng/µL) but also in the DM (0.0008–0.0009 mg SDS/mg of dry DM mass). Yet no reference threshold exists in the literature [41]. Hou et al. were the only ones to use a perfusion-decellularization technique with SDS in a rabbit model [60]. Their protocol consisted of rinsing with heparinized buffer with adenosine, followed by 16 h of 1% SDS perfusion via the common carotid artery, rinsing, and then 15 min of Triton X-100 treatment and a final rinsing. The perfusion pressure was maintained at 105.5 mmHg. Not only was their protocol short (65 h), but they reached all three of Crapo’s criteria, confirming the effectiveness of a perfusion-decellularization model. Bhrany et al. chose to prevent ECM degradation with a cocktail of protease inhibitors including 0.2 mM phenylmethylsulfonyl fluoride, EDTA 5 mM disodium salt, 10 M leupeptin, and 1.5 M pepstatin [52]. After cell lysis, the intracellular proteases are released and could affect the ECM [37]. Nevertheless, the exact advantage of these protease inhibitors has not been systematically evaluated. Sitthisang et al. compared different SDS concentrations—varying from 0.1, 0.25, 0.5, and 1%—with decellularization by cannulation for up to 14 days in a pig model. The aim was to find the ideal protocol with the lowest SDS concentration and the shortest duration while achieving effective decellularization. They found that an SDS of 0.1% was unable to reach this goal, even after 14 days. The other concentrations allowed for complete decellularization at day 3 (1%), day 4 (0.5%), and day 5 (0.25%), so they looked at the preservation of the ECM (collagen type IV and laminin). While the collagen was well preserved, the laminin was not observed anymore by Immunohistochemistry (IHC) with the 1% SDS condition. They finally selected five days of 0.25% SDS for their protocol with an effective decellularization (36 ± 12 ng dsDNA/mg) [44]. The team of Barbon et al. compared three different protocols using 4% SDC, 0.002% SDS, or 2% Tergitol™, a non-ionic detergent. They found that each protocol reached an amount of residual dsDNA inferior to 50 ng/mg but on wet tissue, with a significantly lower level with the SDC [40].

**Octylphenoxypolyethoxyethanol**—Triton X-100, as the Tergitol™, is a non-ionic detergent able to disrupt lipid–lipid, lipid–protein, and DNA–protein interactions and, to a lesser extent, protein–protein interaction. However, as non-ionic detergents are insufficient to remove nuclei and DNA, they cannot be used alone [64,74,75]. Ozeki et al. compared two different protocols: the first one using 4% deoxycholic acid (DEOX, anionic detergent) followed by DNase-I; the second one using 1% Triton X-100 with EDTA, DNase-I, and RNase-A. They both eliminate all cell nuclei, as revealed by classical histology but with a residual quantity of dsDNA superior to 50 ng/mg, which is significantly lesser with DEOX. The DEOX protocol was also preferred because of a clear macroscopic alteration of the esophageal texture with the Triton X-100 in this experiment [51].

**Soap Nut Pericarp Extract**—SPE was an innovative detergent used by the team of Goyal et al. [49]. They chose the fruit of *Sapindus mukorossi*, a member of the family *Sapindaceae*, containing 10.1% saponins in the fruit pericarp (natural non-ionic surfactant). Soap nut pericarp contains triterpenoidal saponins: oleanane, dammarane, and tircullane [93]. They tested different treatment durations and concentrations with agitation at RT in a caprine model. It appeared that 72 h with 5% SPE achieved the best DNA removal (2.70 ± 0.6 ng/mg dsDNA on wet tissue) while preserving the ECM (no significant difference in collagen quantification).

**Biological Agents**—Nucleases (e.g., DNase and RNase) or proteases (e.g., trypsin) have the advantage of specificity for biologic substrates. Nucleases cleave nucleic acid sequences, helping with nucleotides removal after cell lysis. They are generally used after detergents that increase internal spacing and porosity, making them easier and quicker to infiltrate into tissues and allowing for the completion of decellularization. However, extensive washing is necessary because residual enzymatic products may invoke an immune response and hamper future recellularization and implantation [37,64,74]. In this review, DNase-I was widely employed with other detergent(s) in 20 of the 26 protocols and RNase-A in combination with DNase-I in 3 protocols. There is significant heterogeneity in the units used in the articles, ranging from U/mL to KU/mL and mg/mL. In 8 out of 20 articles using DNase, the concentration is not mentioned. Trypsin is a serine protease commonly used for its capacity to detach cells from the proteins of the ECM. Nonetheless, its action is time-dependent, and long exposure to trypsin may disrupt collagen and elastin, leading to damages in the ECM and the alterations of mechanical properties [37,64,74]. Only Barbon et al. used trypsin in two decellularization protocols, together with ammonium hydroxide and detergents such as SDS or Tergitol™, with good results [40]. A special consideration when using trypsin is the potential activity inhibition by natural protease inhibitors released from lysed cells [94].

### 4.4. Disinfection and Sterilization

Disinfection is the process of killing or removing all kinds of pathogenic microorganisms except bacterial spores, while sterilization is the process of killing or removing all microorganisms including bacterial spores. The esophagus has a microbiota and is in constant exchange with the extracorporeal environment and nonsterile material such as food. A decellularized esophageal graft should therefore be sterile before clinical applications, but disinfection may be sufficient for experimental purposes. The most common and approved sterilization techniques are **Ethylene Oxide** (EO) or **irradiation** (gamma ray produced by ^60^Co device or electron beam produced by electron accelerator). Other sterilization and disinfection methods comprise **peracetic acid**, alcohol, antibiotics, antimycotic, or sodium azide. The ideal sterilization or disinfection of the DM must remove all of the microorganisms while being non-cytotoxic and preserving its biomechanical properties. Classically, irradiation denatures key structural proteins of the ECM such as collagen, resulting in mechanical alterations, and EO is toxic and its residues cause host-adverse reactions [95]. In this review, one team used EO [46] and four groups chose irradiation [29,30,47,54]. Under certain conditions, PAA can achieve a sterilization effect. Hodde et al. even demonstrated the capacity of PAA (with ethanol) to inactivate the porcine virus in a porcine small intestinal submucosa model [96]. The decomposition products of PAA are non-toxic, but its acidity could affect the physical and chemical properties of the material. Only Nayakawde et al. and Barbon et al. used PAA in their protocols [40,43]. The vast majority of the protocols included **antibiotics and/or antimycotics** (19/26 protocols). There was a large panel of molecules such as penicillin, streptomycin, gentamycin, clindamycin, vancomycin, and amikacin for the antibiotics and Amphotericin B as the antimycotic. However, only the group of Arakelian et al. performed sterility tests because they wanted to develop a clinical-grade decellularized esophagus [32,41,63]. Interestingly, they only used a strong cocktail of antibiotics and antimycotics at the initial decontamination step: an overnight bath of gentamycin, clindamycin, vancomycin, and Amphotericin B under constant agitation. The rest of the process was carried out under sterile conditions in a laminar flow hood and in a bioreactor (reducing open and manual manipulations). Even if some microorganisms were still present in a few samples after the decontamination step, they were all sterile at the control after the decellularization process. This process is already used in other tissues such as human skin allograft [97]. Moreover, Arakelian et al. firstly used activated Amberlite^®^ XAD16N resin (Sigma, L’Isle D’Abeau Chesnes, France)—an ion-exchange resin—to eliminate residual detergent (SDS) and antibiotics in their protocol. Afterwards, Godefroy et al. used, for the same purpose, an activated charcoal cartridge—commonly used for toxic elimination in intensive care units—the Adsorba 300C (Baxter, Deerfield, IL, USA) cartridge. Otherwise, supercritical carbon dioxide is currently explored as an alternative with promising results both for decellularization and for sterilization [98,99].

### 4.5. Composition of the Extracellular Matrix

The extracellular matrix is a well-arranged 3D network divided into a pericellular matrix, allowing for cell attachment and an interstitial matrix providing tissue integrity. The ECM owns structural and functional roles in the regulation of cellular activity and in tissue remodeling and organization. It is composed of collagens, elastin and elastic fibers, laminins, fibronectin, proteoglycans (PGs), glycosaminoglycans (GAGs), and other proteins/glycoproteins [100]. Important trace matrix proteins such as vitronectin, osteopontin, decorin, biglycan, thrombospondin, and many others are less assayed and difficult to quantitate. It is currently unknown what specific molecules are essential for the ideal DM graft, and a wide, quantitative exploration of what remains within a DM is needed [65]. Parker et al. showed that the ECM source is even more important for the cell phenotype than the cell source for the fibroblast gene expression in an idiopathic pulmonary fibrosis model [101]. The exploration of the ECM composition is commonly performed by classic histology, specific IHC, ELISA techniques, or mass spectrometry. The choice of the technique and the researched component is arbitrary and may be variable in assay quality. The unexplored ECM constituents could then lead to potential confirmation bias [65]. Because classical histology is often performed but too subjective, we focused on the authors who quantified the ECM and native components by different techniques: six dosages of collagen, one for the laminin, three for the elastin, one for the fibronectin, and ten for the GAGs. When crucial ECM components—such as collagen, elastin, and GAGs—are damaged, it could greatly alter the biomechanical properties of the matrix, hampering its use as a scaffold.

The **GAGs** are pivotal for binding growth factors—such as fibroblast growth factor (FGF), vascular endothelial growth factor (VEGF), and platelet-derived growth factor (PDGF)—and cytokines, and they retain water in the tissues, which contributes to the viscoelastic properties. The main GAGs found in the esophagus are hyaluronic acid (un-sulfated GAG), dermatan sulfate (DS), and heparan sulfate (HS), then lower amounts of chondroitin sulfate (CS) and their over-sulfated forms, whereas heparin was not detected. They are found in the ECM, in intracellular vesicles, and on the cell surface via the PGs [63,102]. A systematic decrease in the amount of GAGs was reported in all ten protocols where a quantification was performed [29,30,40,41,42,43,46,53,55,59]. It could be explained by the use of ionic detergents (like SDSs that can harm the GAGs) but also by the removal of the cells themselves because of the presence of GAGs on their surfaces [37,64,74]. The team of Barbon et al. explored three different decellularization protocols and observed a better preservation of GAGs with the SDS (82%) and Tergitol™ (92%) than with the SDC (70%) [40].

**Collagens** are a main constituent of the ECM (over 30%), procuring stability, elasticity, and providing frames for the anchoring of multiple proteins. **Elastin**—composing the elastic fibers—is essential for the ECM to perform reversible and repetitive deformation, which is a major activity of the esophagus. Collagens and elastins are the most abundant and primary structural elements of the ECM. They may support tissue stretching when PGs and GAGs can resist opposite compression forces [43,65,66,100]. Globally, most of the decellularized esophagi maintained their collagen levels while only two of the six performing quantifications showed a decrease [43,59]. Furthermore, two teams evaluated the collagen content using a quantification of the hydroxyproline—present almost exclusively in collagen. Mallis et al. and Barbon et al. showed no significant difference between the native esophagus and the DM [40,53]. Nonetheless, Barbon et al. observed a better preservation of the hydroxyproline with the SDS or the Tergitol™ than with the SDC. Some protocols showed an increased collagen content after decellularization that can be explained because cells and possibly some proteoglycans no longer contribute to the dry weight [30,40,42]. For the elastin, the same observation of preservation was made in the three protocols quantifying it [41,43,59].

**Fibronectin** regulates mechanical properties but also interacts with integrins, among others, regulating cellular adhesion. Its preservation has a major role when considering recellularization. **Laminins** are a pivotal constituent of the basal membrane (BM), together with the collagen. They possess a structural role but can also interact with surface cell receptors and influence cellular differentiation [66,103]. Orozco-Vega et al. were the only group to quantify the laminin and fibronectin contents in the decellularized submucosa matrix of pigs and piglets. They noticed a significant decrease in the fibronectin content in the pig and piglet esophagus and a significant decrease in the laminin in the piglets only, while it was very well preserved in the adults [46].

### 4.6. Biomechanical Test

One way to indirectly explore the ECM composition is testing the biomechanical properties of the scaffold, which greatly depends on the collagens, elastins, and laminins. It will confirm the capacity of the ECM to resist long-term mechanical stress, as during deglutition in physiologic conditions. Considering a potential graft, it could also reduce the risk of stenosis/perforation. Moreover, the stiffness of the matrix could also influence the future recellularization by the mechano-transduction process [104]. Yet, most of the studies only explore an esophagus strip, composed of all the layers or only one. This is technically easier, but it does not reflect the 3D architecture of the esophagus and its mechanical behavior, depending on the interaction between the muscle, submucosa, and mucosa layers [105]. That is why we only consider the biomechanical test on tubular esophageal segments here. However, the esophagus must allow radial deformation for the passage of an alimentary bolus, while its longitudinal deformation remains quite limited. This absence of longitudinal elongation is the very essence of the problem in esophageal reconstruction. The radial deformation, often represented as the burst pressure, therefore seems more pertinent than the radial elongation.

In this review, only 14 authors explored the biomechanical properties of the DM, and only four with a tubular segment. Bhrany et al. explore the burst pressure of the rat esophagus after SDS and observed a significant decrease, even if they consider this result not clinically relevant given that the maximal pressure was 10 times the normal intraluminal rat esophageal pressure [52]. Hou et al. did not find any significant difference in tensile strength for the maximal elongation between the native and decellularized rabbit esophagus after SDS [60]. Goyal et al. found significant alterations of the DM with SPE in their caprine model: a reduction in the stress and strain but an increase in the elastic modulus (rigidity) [49]. Anisotropic behavior was observed by Luc et al., with a significant rise in the elastic modulus for the longitudinal axis (with a significant strain reduction and stress increase) and the opposite for the circumferential direction. The burst pressure could not be reached, linked to the porosity of the DM (liquid leakage). They used an SDC-based protocol in the pig model [29].

Globally, the matrix seems stiffer after decellularization, even if it can deform more circumferentially. This could be explained by the decellularization itself, with the removal of the smooth muscle cells contributing to the biomechanical properties of the organ. More studies are needed to better characterize the decellularized esophagus and its future in vivo transplantation.

### 4.7. Cytotoxicity, Biocompatibility, and Graft Assay

The first widely accepted definition of biocompatibility, dated in 1986, is “the ability of a material to perform with an appropriate host response in a specific application” [106]. Presently, biocompatibility can be assessed using ISO standard procedures (International Organization for Standardization) to manage the biological risk of medical devices. With the ISO 10993—biological evaluation of medical devices—the graft can be tested for extractable components and local or systemic effects in vitro (cultured cells) and in vivo (living research animals). In most cases, a material with no measurable extractables will be considered biocompatible [107]. The cytotoxicity test is included in the biocompatibility evaluation and is designed to determine the biological response of mammalian cells in vitro using appropriate biological parameters. It is described in ISO 10993-part 5 [108]. Concerning the in vivo effects, the host reaction to the implantation of the device in soft tissues is the most used method. It can be explored with ISO 10993-part 6 [109]. The latter is far more complex due to numerous factors depending on both the host and the DM, such as the choice of the animal species, the anatomical site of the graft, the timing of explantation, and the metrics used to assess the host responses [65]. After implantation, macrophages play versatile roles in determining the fate of the DM graft. They can display an inflammatory phenotype (M1) with the production of inflammatory cytokines such as IL-1ß, IL-6, and TNF-α but also induce Th1-type inflammatory responses. On the other hand, polarization into M2 macrophages can facilitate tissue repair and constructive remodeling and induce Th2 response. Classically, the presence of cellular material promotes M1 macrophages while an effectively decellularized matrix favors M2 macrophages. Actually, macrophages are able to modify their polarization depending on local stimuli during wound healing—which involves adaptive immunity and triggers remodeling—resulting in an M2/M1 ratio [47,66,68,110]. The DM has specific sites for cellular adhesion, but the decellularization-mediated changes in the ECM ultrastructure activate a group of potential matrikines—peptides liberated by partial proteolysis of ECM macromolecules, which are able to regulate cell activities—such as collagen IV, integrin, and the C terminal end of globular laminin [61,66,111]. These matrikines should be explored further to address the graft-specific host immune response and select appropriate criteria. Another well-known antigen is the α-gal epitope—abundantly present on the cell surface of nearly all species except humans and Cercopithecoidea (old world monkeys)—which should be a matter of concern because of the hyperacute and acute immune rejection of the decellularized xenograft. However, like for the HLA previously discussed, the precise threshold of the α-gal epitope to induce rejection is currently missing [61,65].

In this review, 16 authors explored the cytotoxicity of their DM through different qualitative or quantitative tests with satisfactory results. Four teams used a widely used assay—the MTT or the XTT cytotoxicity assay—to quantify the cell viability, and they all observed the absence of deleterious effects from the DMs [29,40,55,57]. Two other teams performed cytotoxicity analyses on Balb/3T3 cells—another validated tests according to ISO 10993-part 5—and confirmed the absence of cytotoxicity induced by the DMs [41,63]. Concerning biocompatibility, there were also a wide variety of tests and analyses in the 12 investigations. The implantation site could be subcutaneous in the back, the abdomen, or the neck but also intraabdominal inside the great omentum. The animal models were mice, rats, or pigs, and the extraction timing varied from 7 days to 6 months. The extracted DM was investigated through classical histology in order to assess the host immune response with observations such as the size and number of vessels or the type and number of inflammatory cells. Koch et al. and Saleh et al. investigated the ratio of M1/M2 macrophages in classical or modified esophageal DMs but only Saleh et al. compared the classical esophageal DM to the native esophagus [47,55]. They found a lower M1/M2 macrophage ratio in esophageal DM. Interestingly, Luc et al. and Levenson et al. used a semiquantitative histomorphology analysis including several criteria: cellular infiltration, presence of multinucleate giant cells, vascularization, connective tissue organization, encapsulation, and DM degradation. Higher scores indicate constructive remodeling response, while low scores refer to scar tissue or foreign body type responses [112]. Lenvenson et al. observed a score of 10 after 14 days in the great omentum—classifying the matrix favorable to tissue remodeling—while Luc et al. reached a good score of 12 at two weeks in the abdominal subcutaneous fat but falling to 8 at day 35 [29,32]. Melkonyan et al. decided to explore the serum cytokine levels of rats after the subcutaneous implantation of the native esophagus or DM, with a sham and a control group. They found a decrease in the IL-1α, IL-4, IL-17A, and IFN-**γ** concentration on the 21st day after DE implantation compared to native ones, concluding there were “positive dynamics of the wound healing process and the absence of local processes of inflammation and rejection of the decellularized matrices”.

Four teams attempted the graft of their circumferential full-thickness esophageal DM in an animal model (three pigs and one rabbit) [29,30,31,32]. Only the studies of a non-recellularized matrix were considered. This was marked by a high rate of stenosis/leakage-related complications.

### 4.8. Storage

The rapid evolution of tissue engineering to preclinical and clinical assay leads to the need for a storage solution for the scaffold. Moving from bench-to-bedside requires a ready-to-use product with safe transport and bio-banking. Urbani et al. and Godefroy et al. were the only ones to explore the cryopreservation of the DM, using a well-known protocol with dimethyl sulfoxide (Me_2_SO) coupled with slow cooling down to –160 °C [41,59]. Me_2_SO is a cell-permeating agent reducing the rates of ice nucleation and crystal growth and protecting proteins from denaturation through electrostatic interactions. The team of Urbani et al. explored the storage of the scaffold under two different conditions: the slow cooling medium (SCM) versus 4 °C in PBS. They compared the storage durations of 2 weeks, 4 weeks, and 3 and 6 months. For the short-term period (2 and 4 weeks), 4 °C in PBS is sufficient, but clear degradation of the matrix is observed for longer durations. Conversely, the SCM protocol represents an effective technique to preserve the DM, confirmed by histology, ECM component quantification, scanning electron microscopy (SEM), and biomechanical testing. Godefroy et al. tested the DM after 3 weeks and confirmed the same conclusion for this short period (preservation of the histology, structure at SEM, and biomechanical properties).

## 5. Conclusions

The present study has some limitations. First, only 26 articles were included in this systematic review despite an extensive initial screening. Moreover, the animal models, the decellularization protocols, and the obtention of the outcomes were very heterogeneous. This led us to perform a systematic narrative synthesis instead of a meta-analysis. Afterward, because of the wide variety of potential DMs, their use, and site of implantation, it is currently difficult to define the expected properties of such a graft in each situation. A better understanding of which molecules are important to preserve in the ECM and how the host tissue reacts to the DM is required, among other things. Without further standardization of decellularization protocols, it would be difficult to imagine the production of large-scale DM for clinical purposes. There are a lack of studies evaluating and comparing different esophageal decellularization protocols with the same detergent in terms of concentrations, cycles, temperature, etc. Despite a large number of protocols, they often seemed to be very empirical. Even the optimal animal model is not determined yet, although the pig seems to be the best choice and the most used one. However, SDC and SDS confirmed their preference and efficacity to decellularize the esophagus in this review. Other detergents or methods are likewise promising, such as SPE, supercritical carbon dioxide, or apoptosis-assisted decellularization.

Alternatives to current surgical strategies for esophageal reconstruction are needed because of high post-operative morbimortality, even in expert centers. On the other hand, a DM remains useless by itself for any clinical reconstructive application. Beyond decellularization, which is, as shown here, nearly completely mastered, the achievement of a reliable recellularization process remains the key to unlock any anatomical and functional bioconstruction of a transplantable and vascularized esophageal conduit able to provide better outcomes for esophageal surgery.

The obtention of a decellularized esophageal graft seems to be a topic of interest since 21 of 26 articles were published in the last 9 years. This systematic literature review shows that decellularization of the esophagus is feasible, with at least the absence of visible cell nuclei and a satisfactory conservation of the ECM, even if the GAGs often suffer a loss. The optimal protocol is difficult to define because of the important variability of animal models, protocol characteristics, and outcome measurements.

## Figures and Tables

**Table 1:** Literature search strategy.

Database	Search Strategy
Embase	(‘esophagus’/exp OR ‘esophagus’:ti,ab,kw OR ‘esophageal’:ti,ab,kw OR ‘oesophagus’:ti,ab,kw OR ‘oesophageal’:ti,ab,kw OR ‘esophagi’:ti,ab,kw OR ‘oesophagi’:ti,ab,kw ) AND (‘decellularization’/exp OR ‘bioengineering’/exp OR ‘tissue scaffold’/exp OR ‘extracellular matrix’/exp OR ‘decellularization’:ti,ab,kw OR ‘decellularized’:ti,ab,kw OR ‘tissue engineering’:ti,ab,kw OR ‘tissue-engineered’:ti,ab,kw OR ‘extracellular matrix ’:ti,ab,kw OR ‘decellularisation’:ti,ab,kw OR ‘decellularised’:ti,ab,kw OR ‘de-cellularised’:ti,ab,kw OR ‘de-cellularized’:ti,ab,kw )
Pubmed	(“Esophagus”[mh] OR “esophagus”[tiab] OR “esophageal”[tiab] OR “oesophagus”[tiab] OR “oesophageal”[tiab] OR “esophagi”[tiab] OR “oesophagi”[tiab] ) AND (“Extracellular Matrix ”[mh] OR “Tissue Scaffolds”[mh] OR “Bioengineering”[mh] OR “decellularization”[tiab] OR “decellularized”[tiab] OR “tissue engineering”[tiab] OR “tissue-engineered”[tiab] OR “extracellular matrix ”[tiab] OR “decellularisation”[tiab] OR “decellularised”[tiab] OR “de-cellularised”[tiab] OR “de-cellularized”[tiab] )
Scopus	(KEY ( {esophagus} ) OR TITLE-ABS ( {esophagus} ) OR TITLE-ABS ( {esophageal} ) OR TITLE-ABS ( {oesophagus} ) OR TITLE-ABS ( {oesophageal} ) OR TITLE-ABS ( {esophagi} ) OR TITLE-ABS ( {oesophagi} ) ) AND (KEY ( {extracellular matrix} ) OR KEY ( {tissue engineering} ) OR KEY ( {Tissue Scaffolds} ) OR KEY ( {Cell Engineering} ) OR KEY ( {bioengineering} ) OR KEY ( {decellularization} ) OR KEY ( {tissue scaffold} ) OR KEY ( {decellularized extracellular matrix} ) OR TITLE-ABS ( {decellularization} ) OR TITLE-ABS ( {decellularized} ) OR TITLE-ABS ( {tissue engineering} ) OR TITLE-ABS ( {tissue-engineered} ) OR TITLE-ABS ( {extracellular matrix } ) OR TITLE-ABS ( {decellularisation} ) OR TITLE-ABS ( {decellularised} ) OR TITLE-ABS ( {de-cellularised} ) OR TITLE-ABS ( {de-cellularized} ) )

**Table 2:** Decellularization protocol details of the included studies.

Reference	Animal Model: Strain, Sex, Age, Weight, Number	Decellularization Protocol (DP): Chemical and Biologic Agents	DP: Physical Method(s)	DP: Temperature(s)	Disinfection/Sterilization
Ozeki et al. (2006) [51]	Wistar rats, adult, *n* ≥ 12	Po 1: 1% sodium azide + PBS (12 h) 4% DEOX + sodium azide (24 h); 1 M NaCl + 0.2 mg/mL DNase I + PBS (12 h) Po 2: 1% TRITON X-100 + 0.02% EDTA + 20 mg/mL RNase A + 0.2 mg/mL DNase I + PBS (72 h)	Agitation	RT, 37 °C	AB/AM at every step (PSA); sodium azide (1%)
Bhrany et al. (2006) [52]	Fischer 344 rats, *n* = 17	hypotonic 10 mM Tris buffer + EDTA 5 mM + 10 M leupeptin + 1.5 mM pepstatin (48 h); 1% Triton-X 100 + Tris buffer (48 h); Sorensen’s PB (3 h), DNase I (400 U/mL) + RNase-A (0.125 mg/mL) (6 h); PBS (30 min); 0.5% SDS + Tris buffer (24 h); PBS (24 h)	Agitation	RT, 4 °C, 37 °C	AB/AM at every step (PSA); 70% ethanol (final, 12 h)
Mallis et al. (2018) [53]	Sprague-Dawley rats, 250–300 g, *n* = 30	CHAPS buffer (8 mM CHAPS + 1 M NaCl + 25 mM EDTA)(6 h); SDS buffer (1.8 mM SDS + 1 M NaCl + 25 mM EDTA in PBS) (6 h); α-Minimum Essentials Medium (α-MEM) + 40% *v*/*v* Fetal Bovine Serum (FBS) (6 h)	Agitation (350 rpm)	RT, 37 °C	nothing or n.r.
Urbani et al. (2018) [54]	Sprague-Dawley rats, male, 200–300 g, *n* = 6	2 cycles of (deW (24 h); 4% SDC (4 h); 2000 KU DNase-I + 1 M NaCl (3 h))	Cannulation (1 mL/min)	RT, 4 °C	AB/AM initial step (PSA), gamma irradiation (final, dose not referred)
Saleh et al. (2019) [55]	Sprague-Dawley rats, male, 8 weeks	PBS (30′); 0.1% SDS (6 h); PBS (2 h)	Cannulation (5 mL/min)	n.r.	nothing or n.r.
Melkonyan et al. (2020) [56]	Wistar rats, male, 230 ± 50 g, *n* = 55	deW (1 h); 4% SDC + EDTA (3 h); PBS (10′); 2000 IU/mg DNase-I + PBS (1 h); PBS + AB (24 h)	Cannulation (6 mL/min)	RT	AB initial and final step (PS)
Eftekharzadeh et al. (2022) [58]	Sprague-Dawley rats, adult, 200–250 g, *n* ≥ 12	diW (48 h); PBS + heparin (1 h); 0.5% SDS + deW (20 h); deW (1 h); Triton X 1% + deW (1 h), PBS (20 h)	Cannulation (6 mL/min)	n.r.	AB/AM initial and final step (PSA); Gentamycinat by cannulation before decellularization
Kuevda et al. (2018) [57]	Wistar rats, male, 220 ± 50 g, *n* = 30	4% SDC, purified water, porcine pancreatic DNase I, PBS (21 h of decellularizing solutions, no further details)	Cannulation (6 mL/min)	n.r.	nothing or n.r.
Urbani et al. (2017) [59]	Syngeneic New Zealand White rabbits, male, 2.0–2.5 kg, *n* ≥ 20	2 cycles of (deW (24 h); 4% SDC (4 h); 2000 KU DNase-I + 1 M NaCl (3 h))	Cannulation (1 mL/min)	RT, 4 °C	AB/AM initial step (PSA)
Hou et al. (2021) [60]	New Zealand rabbit, 12 weeks, 2–2.2 kg, *n* ≥ 18	PBS + heparin + adenosine (15 min); 1% SDS + deW (16 h); deW (15 min); 1% Triton-X100 + deW (30 min)	Vascular perfusion (105.5 mmHg)	n.r.	AB final step (PS)
Goyal et al. (2022) [49]	Caprine model, *n* = 6	0.25% EDTA + PBS (4 h, with AB); 100 mL of 5% extract of soap nut pericarp (SPE) (72 h)	Agitation	RT	AB initial step (Amikacin)
Totonelli et al. (2013) [42]	White domestic piglets, 12–16 weeks, 25–40 kg, *n* = 4	3 cycles of (deW (24 h); 4% SDC (4 h); 2000 KU DNase-I + 1 M NaCl (3 h))	Cannulation (0.6 mL/min)	RT, 4 °C	AB/AM initial step (PSA)
Luc et al. (2018) [29]	Domestic pigs, 8–12 weeks, 45–55 kg, *n* = 186	water purified by reverse osmosis + sodium azide (12 h); PBS (6 h); 4% *w*/*w* SDC + sodium azide (24 h); PBS (6 h); 2000 KU DNase-I (5 KU/mL)(12 h)	Cannulation (70 mL/min)	RT	AB/AM initial step (PSA) + sodium azide (0.9 and 0.09%); gamma irradiation (final, 25 kGy)
Arakelian et al. (2019) [61]	Large white/landrace pigs, 8–10 weeks, 25–30 kg, *n* = 10	2% SDS + 5 mM EDTA (72 h); hypotonicW (6 h); activated Amberlite^®^ XAD16 N resin (72 h); 50 mL of 100 U/mL DNase (3 h)	Cannulation (27 mL/min) + Agitation + rotation (5–27 rpm)	RT, 37 °C	AB/AM initial step (Gentamycin, Clindamycin, Vancomycin, and Amphotericin B) + aseptic control
Nayakawde et al. (2020) [43]	Swedish domestic pigs, 45–50 kg, *n* = 25	diW (24 h)—po1: 3 cyles of 4% SDC (4 h); PBS (1 h); 2000 U DNase-I (3.7 U/mL) (3 h); diW (24 h)—po2: 11 cycles of 6% Tri-n-Butyl phosphate (TnBP) (4 h); 6% Triton X-100 (3 h); 2000 U DNase-I (3.7 U/mL) (3 h); diW (24 h)—po3: 11 cycles of ice-cold PBS + 3 cycles of ultrasonication; 2000 U DNase-I (3.7 U/mL) (3 h); DiW (24 h)	Cannulation (3 mL/min) + Freeze/thaw (−80 °C)	RT, 37 °C	AB/AM initial step (PSA); final: 0.18% PAA (3 h); 70% ethanol (5′)
Sitthisang et al. (2020) [44]	Adult pigs, 6 months, 60–90 kg	0.25% SDS *w*/*v* solutions (best compared to 0.1–0.5–1%)(from 3 to 14 days, final 5 days (=120 h)); deW (24 h)	Cannulation (0.1–0.2 mL/min)	n.r.	AB initial and final step (PS)
Hannon et al. (2022) [30]	Piglets, male and female, 1–8 days, 1–2.5 kg	deW (48 h); 2 cycles of 4% SDC (4 h)—deW (overnight)—2000 KU DNase-I (11.1 KU/mL) (3 h)—deW (overnight); deW (48 h)	Cannulation (1–3 mL/min)	RT	gamma irradiation (final, 1.8 kGy, 72 h)
Marzaro et al. (2022) [31]	Sus scrofa domesticus pigs, adult, 40 kg, *n* = 10	Ultrapure water + AB (48 h); 5 cycles of 4% SDC (4 h); 2000 KU DNase-I in 1 M NaCl (3 h); ultrapure water	Cannulation + Freeze/thaw (−80 °C)	RT, 4 °C, 37 °C	AB initial step (PS); ethanol (final, increasing percentages)
Orozco-Vega et al. (2022) [46]	Piglets, 1–21–45 days and adults	diW, 0.8% CaO/1% Triton X-100 (overnight); 1% (NH_4_)_2_SO_4_ (2 h); PBS (30 min); 4% SDC (3 h); 1%Triton X-100 (3 h); PBS (overnight); 4% SDC (3 h for eso Day 1 and 5 h for eso Day 21–45); PBS (overnight); Tris-HCl + DNase-I 0.2 mg/mL + RNase-A 0.02 mg/mL (3–4–6 h for eso Day 1–21–45), EDTA (30′); sterileW (48 h)	Cannulation + Freeze/thaw (−70 °C)	RT, 4 °C	EO (duration not referred)
Levenson et al. (2022) [32]	Hybrid landrace, large white, and Pietrain pigs, female, 4 months, 50 kg, *n* = 18	2% SDS + 5 mM EDTA (72 h); hypotonicW (6 h); activated Amberlite^®^ XAD16N resin (72 h); 50 mL of 100 U/mL DNase (3 h)	Cannulation (27 mL/min) + Rotation (5–27 rpm)	RT, 37 °C	AB/AM initial step (Gentamycin, Clindamycin, Vancomycin, and Amphotericin B) + aseptic control
Koch et al. (2012) [47]	Deutsche Landrasse pigs, 25–65 kg, *n* = 50	0.9% NaCl; 5% SDS (7 days—168 h); PBS (48 h); DNase 200 µg/mL (12 h)	Bathing	RT, 37 °C	Gamma irradiation (final, 25 kGy)
Marzaro et al. (2006) [45]	Pigs, 3–4 days	5 cycles of diW (72 h); 4% SDC (4 h); 2000 KU DNase-I in 1 M NaCl (3 h)	Bathing	n.r.	AB/AM initial step (molecules not referred)
Zambon et al. (2016) [48]	White domestic piglets, 12–16 weeks, 25–40 kg, *n* = 4	3 cycles of deW (24 h); 4% SDC (4 h); 2000 KU DNase-I (0.46 KU/mL) + 1 M NaCl (3 h)	Cannulation (0.6 mL/min)	RT, 4 °C	AB/AM initial step (PSA) + supercritical CO_2_
Sotnichenko et al. (2016) [50]	Rhesus macaques (Macaca mulatta), male, *n* = 4	po1: 2 cycles of diW (1 h); 4% SDC + EDTA (1 h); PBS (10 min); DNase-I (10 U/mL) (1 h) po2: 4 cycles of 4% SDC + EDTA (1 h); diW (1 h); DNase-I (10 U/mL)(1 h)	Cannulation (po1: 150 mL/min) (po2: 21 mL/min + Freeze/thaw) (−30 °C)	n.r.	Nothing or n.r.
Barbon et al. (2022) [40]	Human, *n* ≥ 3	po1: deW (24 h); 4% SDC (4 h); 2000 KU DNase-I (3 h); deW (overnight); 2 cycles (+72 h AB; 1 h PAA)—po2–3: deW (24 h); 0.05% trypsin-0.02% EDTA (1 h); 0.002% SDS (po2) OR 2% TergitolTM (po3) + 0.8% ammonium hydroxide (NH4OH) (72 h); deW (72 h)	Agitation + Freeze/thaw (−80 °C)	4 °C, RT for po1 (+37 °C for po2–3)	po1: AB initial and final step (PS) + PAA (final, 0.1 M, 1 h)
Godefroy et al. (2023) [41]	Human, *n* = 20	sterileW (3 h); 2% SDS + EDTA (72 h); sterileW (6 h); ion-resine/charcoal (72 h); DNase-I (10 U/mL) (3 h); PBS + EDTA	Cannulation (27 mL/min) + Agitation + rotation (4–27−100 rpm)	RT, 30 °C, 37 °C	AB/AM initial step (Gentamycin, Clindamycin, Vancomycin, and Amphotericin B) + aseptic control

po: protocol; deW: deionized water; diW: distilled water; RT: room temperature; n.r.: not reported; AB: antibiotic; AM: antimycotic; PS: Penicillin Streptomycin; PSA: Penicillin Streptomycin Amphotericin B, and PAA: peracetic acid.

**Table 3:** Decellularization protocol results of the included studies.

Reference	Cell Nuclei	DNA Quantification	Residual DNA Fragments	Collagen	Laminin	Elastin	Fibronectin	GAG	Mechanical Test
Ozeki et al. (2006) [51]	no nuclei (H&E)	DEOX (140 ng/mg) vs. TRITON (2410 ng/mg) (DT)	-	-	-	-	-	-	-
Bhrany et al. (2006) [52]	no nuclei (H&E)	-	-	Q	Q	Q	Q	-	Burst pressure SD
Mallis et al. (2018) [53]	no nuclei (DAPI)	93 ± 26 ng/mg (DT)	-	Q	-	Q	Q	SD (+Q)	-
Urbani et al. (2018) [54]	no nuclei (H&E/DAPI)	120 ng/mg (*) (WT)	-	Q	-	Q	-	Q	Strips
Saleh et al. (2019) [55]	no nuclei (H&E/DAPI)	14 ng/mg (*) (WT)	no	NSD	Q	-	Q	SD	-
Melkonyan et al. (2020) [56]	-	123.85 +/− 22.61 ng/mg (WT)	-	-	-	-	-	-	-
Eftekharzadeh et al. (2022) [58]	no nuclei (H&E)	-	-	-	-	-	-	-	Strips
Kuevda et al. (2018) [57]	no nuclei (H&E)	-	-	-	-	-	-	-	-
Urbani et al. (2017) [59]	no nuclei (H&E/DAPI)	270 ng/mg (2 cycles) (*) (WT)	-	SD	-	NSD	-	SD	Strips
Hou et al. (2021) [60]	no nuclei (H&E/DAPI)	41 ± 7 ng/mg (DT)	no	Q	-	-	-	-	Tensile Strength NSD
Goyal et al. (2022) [49]	no nuclei (H&E/DAPI)	2.70 ± 0.6 ng/mg (WT)	no	NSD	-	-	-	-	Stress and Strain SD EM SI
Totonelli et al. (2013) [42]	no nuclei (H&E)	127 ± 28 ng/mg (WT)	-	NSD	-	-	-	SD	-
Luc et al. (2018) [29]	no nuclei (H&E)	50 ± 18 ng/mg (DT)	no	-	Q	Q	Q	SD	Longitudinal Strain SD, Stress SI, EM SI, Strength SI—Circumferential Strain SI, Stress NSD, EM SD, Strength NSD
Arakelian et al. (2019) [61]	no nuclei (H&E/DAPI)	24 ± 13 ng/mg (WT)	no	Q	-	Q	-	Q	Strips
Nayakawde et al. (2020) [43]	no nuclei (H&E) (po1 only)	96 ng/mg (*) (DT)	-	NSD insoluble collagen/SD soluble collagen (+Q)	Q	NSD (+Q)	Q	SD	-
Sitthisang et al. (2020) [44]	no nuclei (H&E/DAPI)	36 ± 12 ng DNA/mg (DT)	-	Q	Q	-	-	-	-
Hannon et al. (2022) [30]	no nuclei (H&E)	32.6 ng/mg (*) (DT)	-	SI (+Q)	Q	-	Q	NSD (+Q)	Strips
Marzaro et al. (2022) [31]	no nuclei (H&E)	15.83 ng/mg (DT)	-	Q	Q	Q	Q	-	Strips
Orozco-Vega et al. (2022) [46]	no nuclei (H&E)	Day 1: 8.4 ng/mg Day 21: 12.1 ng/mg Day 45: 34.2 ng/mg (*) (DT)	-	-	NSD adult, SD piglet (+Q)	-	SD	SD	Strips
Levenson et al. (2022) [32]	no nuclei (H&E)	-	-	-	-	-	-	-	-
Koch et al. (2012) [47]	no nuclei (H&E)	8.05 ± 2.01 ng/mg (WT)	-	Q	-	Q	Q	-	-
Marzaro et al. (2006) [45]	no nuclei (H&E)	-	-	-	-	-	-	-	-
Zambon et al. (2016) [48]	no nuclei (H&E)	-	-	Q	-	-	-	Q	Strips
Sotnichenko et al. (2016) [50]	no nuclei (H&E)	-	-	Q	Q	-	Q	-	-
Barbon et al. (2022) [40]	no nuclei (H&E)	po1: 15 ng/mg (*) po2: 39.8 ng/mg (*) po3: 44.3 ng/mg (*) (WT)	-	Q	Q	Q	-	SD (po1)	Strips
Godefroy et al. (2023) [41]	no nuclei (H&E)	35.5 ± 19.5 ng/mg (DT)	No	-	-	NSD	-	SD	Strips

DT: dry tissue; WT: wet tissue; SD: significant decrease; SI: significant increase; Q: quality assessment; NSD: no significant difference, (*): data measured with “a ruler for windows”, po: protocol; and EM: elastic modulus.

## Data Availability

No new data were created or analyzed in this study.
